# Supplementary material for: Are carbon emissions trading and green financial instruments synergistic? -Comprehensive quantitative research based on content analysis
Source: PLoS One. 2024 Mar 7;19(3):e0298601. doi: 10.1371/journal.pone.0298601 (PMC10919601; doi:10.1371/journal.pone.0298601)
Supplement: S1 Data — (ZIP) [file pone.0298601.s001.zip › Data/地级市Z_F工作报告.zip!/║┌┴·╜¡/╦1⁄2╤╝╔╜╩╨/└┤╫╘╣1⁄2╓┌║┼í╛┬φ┐╦╩2╛▌═°í┐_2017.docx]

各位代表:

现在，我代表市人民政府，向大会报告工作，请予审议，并请市政协委员提出意见。

一、过去五年工作回顾

过去的五年，我们面对复杂形势和各种挑战，在省委省政府和市委的正确领导下，团结带领全市人民，坚定信心，迎难而上，开拓进取，全市经济社会发展和各项事业取得了可喜进步。

——经济发展健康运行。采取一系列重大有效举措，沉着应对经济下行的严峻考验，确保了经济社会发展总体向好。全市地区生产总值年均增长1.5%；社会消费品零售总额年均增长11%；固定资产投资、一般公共预算收入由降转升，持续增长；规模以上工业增加值降幅收窄43.2个百分点。农业种植结构调整步伐加快，综合生产能力持续增强，粮食总产量连续五年稳定在50亿斤以上。畜牧产业和林业经济发展提速。

——转型发展取得积极成效。所有制结构调整加快，非公经济蓬勃发展，占经济总量的比重已达50%以上。第一、第三产业发展加快，第二产业结构发生积极变化。三次产业结构由31.8：46.4：21.8调整到38：20：42。一批招商引资重点项目建成投产，接续产业不断壮大。累计建设投资5000万元以上产业项目280个。龙煤天泰10万吨芳烃、三聚华本LNG等重大项目的建成，实现了现代煤化工从无到有的历史性突破。建龙钢铁、新时代水泥完成改造升级，传统优势产业得到加强。

——改革开放进一步深化。2014年以来，市政府完成重大改革任务179项。完成了新一轮政府机构改革。下大气力推进行政审批制度改革，最大限度减少审批事项、简化审批流程，行政效能大幅提升。商事登记制度、财政预算体制、公共资源交易体制、行政执法体制等重点领域改革扎实推进。认真贯彻国家“一带一路”战略，积极参与“龙江丝路带”建设，以对俄为重点的对外经贸合作持续深化。

——城乡面貌发生深刻变化。全面推动滨水北城、南市区建设改造，主城区实现新的拓展。扎实推进“三供三治”，启动实施“一创两建”，城市公共服务和管理水平显著提升。累计实施城乡基础设施和公共服务设施项目5300多个。改造棚户区住宅7.6万户、农村泥草房4万户。新建一、二级公路132公里，农村公路193公里，城区道路190.4公里，全市路网体系基本形成。安邦河治理持续推动。寒葱沟水库开始供水。市污水处理厂一、二期建成运行。行政村通村公路全部硬化；农村饮水安全基本实现。

——社会事业全面发展。大力推进义务教育均衡发展，办学条件明显改善。新建、改扩建中小学校、公办幼儿园102所；完成义务教育阶段学校标准化建设138所；“两基”教育获国务院嘉奖。深化医药卫生体制改革，覆盖城乡的公共卫生服务体系基本建成；基层医疗卫生机构全部实行基本药物制度。市县（区）博物馆、文体中心、休闲广场等一批大型公共服务设施投入使用。“平安双鸭山”建设扎实推进，全市安全生产形势总体平稳；群众诉求及时有效解决，信访形势持续好转；着力构建社会治安防控体系，人民群众安全感显著增强。

——人民生活质量显著提高。城镇和农村常住居民人均可支配收入分别比2011年增加了5336元、4059元。全市累计新增就业12.9万人，城镇登记失业率控制在4.3%以内。实现了医疗保险市级统筹。城乡居民社保覆盖面、保障标准和各类救助水平逐年提升。3.6万人实现脱贫。全市用于民生支出441.1亿元，年均增长11.6%。

各位代表，刚刚过去的2016年，是本届政府的收官之年，也是困难突出、矛盾凸显、挑战增多的一年，更是我们在困境中团结拼搏、在艰难中奋力前行的一年。我们认真学习贯彻习近平总书记系列重要讲话特别是对我省两次重要讲话精神，扎实落实省委省政府和市委的决策部署，着力稳增长、调结构、促改革、惠民生，着力应对挑战、解决矛盾、化解风险，着力开拓进取、创新担当、真抓实干，较好地完成了市第十五届人大七次会议确定的各项目标任务。预计实现地区生产总值432亿元，同比增长2.6%；固定资产投资128亿元，增长5%；规模以上工业增加值42.6亿元，降幅收窄0.5个百分点；一般公共预算收入20.9亿元，增长11.5%；社会消费品零售总额128.7亿元，增长8%；城镇和农村常住居民人均可支配收入分别达到22100元、12820元，分别增长4%和5%。

（一）狠抓产业项目建设，推进产业转型升级。以城市转型为目标，以做好“三篇大文章”、做实“四头四尾”为方向，科学谋划储备项目，加快项目落地生成，产业项目带动作用明显增强。引进增量，全市招引产业项目123个，到位资金108亿元。投资500万元以上产业项目开复工126个，完成投资63.97亿元。龙煤天泰10万吨芳烃、三聚华本LNG试生产，宝清万里润达粮食仓储及深加工一期、岭东长山万源矿泉水等项目竣工投产，集贤北大仓现代农业产业园、神华宝清2×60万千瓦电厂、新时代水泥废渣综合利用、建龙干熄焦等项目加快建设。盘活存量，引进黑龙江威克特、深圳高锋科技等企业，盘活启动了集贤金谷特优食用酒精、友谊麦芽厂等停产半停产企业22户。创新机制，建立了产业项目谋划生成机制，成立了14个重点产业项目策划生成领导小组，谋划生成储备项目102个。跟踪线索，初步建立起重点招商项目储备库、PPP项目库和企业家数据库，通过各类招商活动，生成项目线索120多个。

（二）实施创新驱动发展战略，新兴产业不断壮大。高度重视创新驱动的核心推动作用，把加快新技术、新业态、新模式引入和应用作为促进经济转型的重要着力点。电商产业蓬勃发展。成立市电子商务服务中心，创办运营了电商创业园、运营中心和四达颐高电商园，孵化电商企业52个，四达颐高、“六六鸭”微商连锁、尚农电子商务等一批新经营模式不断涌现。电商、微商创业群体快速壮大，全市组团（域内外）2000人以上大型电商、微商群近300个，从事电商、微商行业人员达到万人以上。与新华网和深圳报业集团合作，启动了市综合电商服务平台建设。各县区开始启动电商园区建设。宝清县与阿里巴巴合作“村淘”项目落地，集贤县成为国家电子商务进农村综合示范县。成功举办东北地区首届双鸭山微商峰会暨微商品牌产品推介对接会。电商产业的蓬勃发展，带动了越来越多的本地产品进入全国市场。全市小件快递业务量同比增长111.6%，居全省前列。科技创新务实推进。实施了科技型企业三年行动计划。出台了《关于深化科技改革促进科技成果产业化的实施意见》。与哈工大、黑大、省农科院、大连化物所等20余所高校、科研院所建立了协作关系，达成科技成果转化意向65项，成功转化18项。新增科技型企业30家。金融业稳健发展。各金融机构创新金融服务方式，积极开发新的金融产品，对地方经济发展的支撑作用明显增强。各项贷款余额突破830亿元；存贷比102.2%，居全省第一，高出全省平均水平25个百分点。积极发挥政府基金引导作用，建立了创业就业小额贷款担保基金、小微企业和扶贫贷款风险补偿金、产业发展基金。旅游产业充满活力。基础设施不断完善，新的特色旅游项目和精品线路陆续推出，与上海景域集团、深旅集团、中促旅、中旅联盟等签订了合作协议，“中国·黑土湿地之都”品牌进一步叫响。预计全年旅游收入实现22.5亿元，同比增长31.9%。

（三）优化农业产业结构，加快发展现代农业。以提质增效为主攻方向，通过优化农业结构、转变生产方式、创新经营主体等措施，有效推动现代高效农业发展。优化种植业结构。全市调减玉米76万亩，水稻、大豆、杂粮分别调增15.8万亩、54.9万亩和2.4万亩。大力发展绿色生态农业。落实“三减”示范面积31.2万亩，认证绿色有机面积133.8万亩、绿色农产品品牌25个。大力推广农业技术。测土配方施肥推广面积400万亩，标准化栽培率、良种覆盖率分别达到90%和98%。加快农村改革和农业产业化发展。完成农村土地确权登记的行政村占比达到70%，规模以上农业产业化企业发展到108个，农民专业合作社达到1307个，实施农田水利工程10项，农机总动力达到190万千瓦。着力发展畜牧业。新建和扩建投资500万元以上大型畜牧产业项目7个，预计畜牧业增加值实现57.8亿元，同比增长2%。积极推动林业产业发展。林果、山野菜、林下养殖等林业产业稳步发展，食用菌产业发展出现好势头，四个区均新建了食用菌产业园，岭东花菇生产基地初具规模，建成棚室337栋。加大优质农产品外销力度，线上线下同步推进，农产品“种得好”向“卖得好”转变取得喜人成果。

（四）加大改革力度，汇聚发展活力。全年完成重点改革任务128项。深化行政审批制度改革。市本级行政审批事项由155项精减到115项，全省最少；审批时限由29个工作日压缩到6个工作日，全省最短。完善现代市场体系改革。建立了电子政务平台，市县同步开通了网上审批服务系统；建设了市民服务中心；实施“五证合一、一照一码”商事登记制度改革，企业登记注册时限由5个工作日缩短到2个工作日；整合政府采购、项目招投标、土地招拍挂等职能，建立了公共资源交易平台；整合质监、食药、农产品等检验检测机构，建立了市检验检测中心；基本理顺了区级食品药品和市场监管关系。顺利推进财税体制改革。深化预算管理制度改革，全面规范、公开透明的预算制度建立运行；建立起债务风险管控机制；“营改增”税制改革圆满完成；厂办大集体改革稳步推进。创新推动社会管理体制改革。改革社区管理体制，厘清工作职责，盖章事项由90项精简到8项；建设了城市统一公共应急平台，将87个热线整合梳理后全部并入110指挥中心统一接办。

（五）务实推进双深合作，持续扩大对外开放。双深合作向深层次、宽领域发展。两市组织商务代表团开展了9次互访洽谈，在农产品销售和深加工、粮食仓储、旅游市场开发、电子商务、人才培训以及文化、体育、医疗等领域的合作取得了突破性进展。一批合作项目稳步推进。深粮集团投资的深粮中信粮食基地项目已开工建设，深圳黑土地公司投资的惠远无抗冷鲜猪肉产业化项目已在饶河县完成选址，与深农集团合作组建的黑龙江省海吉星农产品销售公司已组建完成，与深圳贝特瑞公司签订了石墨产业园建设合作协议，与深圳报业集团合作启动建设了双鸭山特色农产品电子商务平台。深圳南山区在我市投资建成了全国最大的气膜冰上体育运动中心，填补了我市空白。我市6家企业在深圳文化产权交易所挂牌。在深农产品销售成效突出。在深圳成功举办了双鸭山优质农产品推介会，与深圳盈盛集团合作建设的深圳市双鸭山农产品交易物流中心正式挂牌，并签订10万吨大米、20万吨玉米销售合同。我市大米、红小豆、胡萝卜等绿色农产品通过农迈天下、海吉星商城采购中心、深圳绿膳谷体验馆等进入深圳市场，倍受市民青睐，双鸭山农产品已在深圳具有很高知名度，大米在深圳打出了“北田”、“冷泥”和“田地冰米”等品牌。对外开放进一步扩大。成功举办了“双鸭山经贸合作企业家恳谈会”，400多名客商和中央、省级媒体来双参会，形成了一批合作成果，进一步扩大了双鸭山的对外影响力；着力推进对俄经贸合作，饶河中俄互市贸易区投入运营；预计全市外贸进出口总值实现1.3亿美元。

（六）加快城乡建设步伐，着力改善人居环境。以“一创两建”为载体，着力在设施配套、环境整治、老区改造、生态建设等方面补短板，城乡环境质量和公共服务水平持续提升。交通网络建设取得历史性突破。通过抢抓机遇，竭力争取，我市被列为牡佳客专主线路主要节点站，七星峰隧道控制性工程全面开工，标志着我市将迈进高铁时代。加快重大交通设施建设，依饶公路双鸭山至集贤段、饶盖公路方盛至夹信子段等国省道改造项目竣工通车。惠及民生的城市设施建设和维修改造取得重要进展。西环路竣工通车；完成市政干道改建和市政设施维修项目50个，改造主城区背街巷道1280条；改造主城区老旧小区24个、老旧供热管网34.6公里，实施供热分户改造109栋，受益群众2万余户；寒葱沟水库至净水厂12公里管道复线竣工，实现了主城区24小时供水，彻底结束了饮用矿井疏干水和分时分片供水的历史。生态文明建设成效显著。突出大气污染和水污染治理，全市空气质量达标325天，饮用水水源地水质达标率100%；淘汰关闭污染企业141家、规范整顿企业356家；污水处理二期投入使用。我市被评为全省首个国家森林城市。

（七）切实强化民生保障，促进社会和谐发展。千方百计筹措资金增加民生投入，全市用于民生领域支出109亿元，比上年增长14%。大力发展均衡教育。投入2.2亿元支持义务教育事业；新增省级义务教育标准化学校6所，改造薄弱学校63所，新建和续建学校、公办幼儿园14所；宝清县通过全国义务教育发展基本均衡县（区）督导评估验收；四个区通过义务教育均衡发展省级建设达标验收。积极推动卫生体制改革及遗留问题解决。启动城市公立医院综合改革试点工作，全面取消药品加成，实行零差价销售，降低医疗服务价格，有效缓解了群众看病贵问题。想方设法筹集资金化解历史遗留问题，启动建设了市精神病防治院、传染病医院遗留工程；化解纠纷，筹措资金，启动并加快市新人民医院续建步伐。加快文体事业发展。举办了首届“中国·双鸭山黑土湿地”摄影大赛和深圳赴双采风文化交流等活动，开展大型群众性文化演出活动90余场；启动了全民上冰雪活动，举办了国际冬泳大赛，冰上体育运动中心和青山滑雪场投入使用。改善群众居住条件。采沉区棚改工程完成21325套建设任务，货币化安置10301套；煤矿棚改工程竣工住宅5041套；改造农村泥草房6345户。民生保障水平持续提高。城镇新增就业2.1万人；完善社会救助体系，发放救助资金4.03亿元；城乡低保救助标准比上年提高6%，临时救助力度进一步加大。加强安全稳定。妥善应对和处置“3·10”事件，积极维护了大局稳定；合理解决群众诉求，通过会办督办等方式化解信访矛盾，信访总量大幅下降；狠抓以煤矿为重点的安全生产，各类生产安全事故同比下降83%；大力推进“平安双鸭山”建设，各类刑事案件发案数下降27.1%。有效应对自然灾害，战胜了45年以来乌苏里江饶河段最大洪水，保障了人民群众生命财产安全。

（八）支持双矿改革发展，促进企业脱困转型。大力开展帮扶救助。深入开展“两进一送”活动，制定了6个专项帮扶方案，124个部门的党员干部与双矿困难职工对接，全力保障双矿困难职工基本生活；建立了双矿困难职工帮扶救助长效机制，9719户15271人纳入救助范围。近两年支持双矿援企稳岗补贴共计1.7亿元。妥善接收安置分流人员。专门成立6个工作推进组和15个国有企业接收分流人员，顺利完成了第一批3500名双矿职工组织化分流工作，转岗人员全部重新上岗；第二批转岗分流工作启动得早，抓得实，成立6个再就业服务中心承接推进，千方百计挖掘和开发就业岗位7727个，目前第二批分流人员4783人已全部转岗到位。顺利完成“两供一业”移交。在完成资产清查、应急维修、政策衔接的基础上，6个独立工矿区和3个职能区协议移交部分在去年12月27日全部实现移交，矿区7万多户居民的供暖、供水和物业管理得到有效保障。积极帮助双矿总医院解决土地证、房产证等遗留问题，保障了晋商联盟收购双矿总医院顺利进行。此外，区域协作力度加大。支持森工转型取得新进展，县场共建取得新成果。

（九）加强政府自身建设，大力提升行政效能。以深入开展“两学一做”学习教育为契机，着力强学习、转作风、提效能、严法纪，政府执行力和公信力明显提升。着力提升政府专业化水平，建立了政府系统常态化学习制度，创办了“双鸭山发展讲坛”，举办了9期专业知识讲座。同时，每次市政府常务会议增加了政策解读内容。自觉接受市人大法律监督和市政协民主监督，人大代表建议和政协委员提案答复率100%。加强法治双鸭山建设，全面启动“七五”普法工作。规范了权力清单和责任清单，完善了政府法律顾问制度。加大政务公开力度，15个重点领域全面公开。实施了公务用车改革。严格“三公”经费管理，市本级“三公”经费支出比上年减少16.7%。严格落实廉政建设“一岗双责”，进一步加大了行政监察和审计监督力度。大力优化发展环境，加大企业投诉事项查处力度，查处破坏发展环境案件73件，化解涉企执行积案253件。

法制建设、行政监察、市场监管、防灾减灾、外事侨务、民族宗教、国防动员、统计监测、文史档案等各项工作及妇女儿童、老龄和残疾人事业都取得了新的进展。

各位代表，过去五年是我市经济社会发展取得可喜成就的五年，是城乡面貌变化最大的五年，是人民群众得实惠最多的五年。这是省委省政府和市委正确领导的结果，是全市人民团结奋斗的结果，是历届政府打下坚实基础的结果。特别是在过去的一年里，我们紧紧依靠市委的正确领导，在市人大、市政协的有效监督和大力支持下，紧密团结全市上下、凝聚各方，形成谋发展、促振兴的强大合力；我们高度聚焦经济建设这个中心，抓根本、打基础、谋长远，紧紧抓住项目建设、改革创新、民生改善、社会稳定等大事要事，以重点方面和关键环节的率先突破带动整体工作跃升；我们敢于直面突出矛盾和棘手问题，不畏难、不回避、不推诿，找准突破口和着力点，持续发力，有效解决了一批经济社会发展难题和历史遗留问题；我们充分发扬务实担当的优良作风，尽职尽责、开拓进取、埋头苦干，努力开创出凝心聚力、共谋发展、创新作为的新局面。

各位代表，双鸭山市经济社会取得的每一个成果，城市面貌发生的每一处变化，百姓生活得到的每一点实惠，无不饱含着方方面面的关心与支持，无不凝聚着全市人民的心血与汗水。在此，我代表市人民政府，向为双鸭山发展贡献出智慧和力量的全市人民致以崇高的敬意！向给予政府工作大力支持的人大代表、政协委员、各民主党派、工商联、人民团体和社会各界人士表示衷心的感谢！向积极参与双鸭山发展建设的驻双中省直单位、驻双部队、武警官兵、公安干警致以诚挚的问候！

二、今后五年的奋斗目标和总体把握

今后五年，是全面建成小康社会的决胜时期，也是我市脱困转型、振兴发展的攻坚时期，有利条件与不利因素并存。不利因素，主要体现在四个方面:从全面建成小康社会的目标看，我市经济实现了筑底回升，企稳向好趋势明显，但支撑较快增长的基础还不牢靠，实现地区生产总值比2010年翻一番的压力很大。从转型发展的要求看，结构性、资源性、体制性矛盾还没有根本破解，传统产业偏大、接续替代产业弱小，转方式调结构的任务艰巨。从发展驱动力看，缺乏科技和人才的支撑，新体制新机制还没有完全建立起来，创新驱动发展的动力不足。从发展“量”和“质”上看，经济总量不大、质量效益不高。以上不利因素是我们的工作差距，但也是我们的工作着力方向和潜力。考虑到不利因素的同时，我们还必须看到，有利条件和优势不可多得。一是有政治优势。党中央、国务院对东北振兴高度重视、深切关心，特别是习近平总书记对我省的两次重要讲话，为龙江振兴发展提供了战略指引，为煤城脱困转型指明了方向、注入了强大动力。二是有政策优势。中央着眼于顶层设计，密集出台了一系列“含金量”很高的政策，推动东北振兴发展，特别是有一些政策相当于为煤城脱困转型“量身定制”。三是有基础优势。我市有优势资源和产业基础，改造升级传统产业、培育壮大新兴产业空间广阔，后发优势明显。四是有氛围优势。全市“上下同欲”、干劲十足，老百姓对实现小康目标的追求更加强烈，广大党员干部带头实现脱困转型的热情不断凝聚，全市人民共克时艰、发奋图强的新动能正在形成。这些有利条件和优势，进一步坚定了我们抓转型、建小康的信心和决心，也进而增强了我们的紧迫感、责任感和使命感。

按照市十一次党代会的部署和要求，今后五年双鸭山发展的指导思想是：高举中国特色社会主义伟大旗帜，全面贯彻党的十八大和十八届三中、四中、五中、六中全会精神，深入贯彻习近平总书记系列重要讲话精神，特别是持续贯彻落实对我省两次重要讲话精神，统筹推进“五位一体”总体布局，协调推进“四个全面”战略布局，牢固树立新发展理念，以创新驱动为引领，以城市脱困转型发展为主线，以“四大产业区”建设为切入点，扎实做好“三篇大文章”，持续改善民生，维护社会健康平稳发展，为实现全面建成小康社会、打造活力幸福秀美文明双鸭山而奋斗。地区生产总值年均增长6%以上，城镇登记失业率控制在4.3%以内，城乡居民收入增长与经济增长同步，实现全面建成小康社会目标。三次产业结构由38:20:42调整为25:30:45。煤与非煤产业占规模以上工业增加值的比重由38:62调整到35:65。非公经济占地区生产总值的比重由50.9%提高到57%。

——全面建成小康社会，加快转型发展步伐，必须加快构建新的产业体系。这是发展的支撑所在。做强资源性产业。实施大项目带动战略，加快建设“煤头化尾”、“煤头电尾”项目，推动煤炭深度开发、高端转化增值，把我市建设成国家级煤化工基地和我省重要的电力生产基地。高起点谋划建设石墨、玄武岩等精深加工项目，大力发展富硒食品，充分释放新资源潜能，形成新的发展动力源。做优农业产业。围绕“粮头食尾”、“农头工尾”，抓好粮食深加工特别是要强力推进玉米深加工，持续推进农业供给侧结构性改革，加快发展优质高效农业，把绿色有机食品品牌打得更响，逐步解决量大链短、附加值低、种强销弱、供求失衡、增收困难的问题，在促进农民持续增收、农业提质增效上取得重大成果。做大新兴产业。以黑土湿地为主打品牌，在“中国·黑土湿地之都湿地旅游带”和“乌苏里江·界江风情旅游带”相结合上做大文章，加快推动旅游产业提档升级，把我市打造成全国黑土湿地旅游和界江旅游目的地。大力发展电商微商产业、“互联网+”产业、健康养老产业等现代服务业。推动生产性服务业向专业化和价值链高端延伸、生活性服务业向精细化和高品质转变。

——全面建成小康社会，加快转型发展步伐，必须全面实施创新驱动发展战略。这是发展的动力源泉。把引进科技成果放在首要位置。通过与省内外高等院校和科研院所建立科技成果产业化创新联盟，引进一批科技成果特别是核心关键技术在双实现产业化，实现科技同经济、创新成果同产业、创新项目同现实生产力的有效对接，加快补齐我市科技创新能力弱的短板。抓创新企业培育。激发企业创新内生动力，推动企业成为技术创新决策、研发投入、科研组织和成果转化的主体，培育一批主业突出、技术先进、竞争力较强的企业，力求培育出若干创新型领军企业。全市新增科技型中小企业100户、高新技术企业10户以上。抓创新平台建设。把创新平台打造成集聚创新要素、生成创新动力、构筑创新优势的重要载体，带动大众创业、万众创新，使创新成为发展的核心力量。

——全面建成小康社会，加快转型发展步伐，必须全面深化改革扩大对外开放。这是发展的关键所在。大力转变政府职能。纵深推进“放管服”改革，深化以商事制度改革为突破口的市场监管改革，大力推进市、县（区）综合行政执法改革，把发挥市场在资源配置中的决定性作用和更好地发挥政府作用有机地结合起来，基本实现政府治理体系和治理能力现代化。推进政资、政事、政企分开。完成事业单位分类改革。完成非政府公共部门改革。深化国资改革，确保国有资产保值增值。引导企业加快形成以提高核心竞争力和资源配置效率为目标的市场化经营机制。深化供给侧结构性改革。加大农业供给侧结构性改革力度，完成农业综合配套改革,培育农业农村发展新动能。持续落实“三去一降一补”重点任务，努力实现供需关系新的动态平衡。统筹推进医药卫生、社会保障、生态建设、环境保护等各领域改革。主动对接“一带一路”战略，融入“龙江丝路带”建设，构建以双深合作为基础，以对俄为重点的全方位对外开放新格局，以开放促开发、以开放促转型。

——全面建成小康社会，加快转型发展步伐，必须增强城市综合承载力。这是发展的重要依托。突出规划引领。坚持全市“一盘棋”，以“五个统筹”为指导，高标准、高质量编制城市建设总体规划和专项规划。完成四个区和集贤县、友谊县一体化规划。强化规划的刚性约束，以规划引领城市科学建设、科学发展、科学管理。加快交通基础设施建设。牡佳客专建成运营，饶河沿边铁路尽快建成，集当公路双鸭山至宝山段、双鸭山至宝清高速公路、双鸭山至饶河一级路改造建成通车，推动佳木斯机场、饶河机场、宝清机场建成通航，形成现代化、立体化的大交通体系，从根本上打破交通瓶颈制约。加大城市基础设施建设力度。建设完成中心站立交桥、建设路立交桥、云峰山隧道。实现四个区和集贤县公交一体化。力争大叶沟水库、“三江联通”工程配建水库——常胜水库建成蓄水，结束城市缺水的历史。实现市区和县城24小时供水，完成主城区天然气置换，城镇清洁取暖、供排水普及率达到100%，燃气普及率达到90%以上，城市功能基本完善。建设生态文明。完成安邦河全流域治理，全市主要河流水质达到Ⅲ类以上。建龙空气污染得到有效治理，城市空气质量优良天数达到350天以上。城市生活垃圾处理工程建成运营，四个区和集贤县、友谊县垃圾实现统一处理，生活垃圾分类及无害化处理率均达到100%。

——全面建成小康社会，加快转型发展步伐，必须切实增进人民群众福祉。这是发展的目的所在。提高人民收入水平。到2020年，城镇和农村常住居民人均可支配收入比2010年翻一番。城乡居民收入差距逐步缩小、中等收入人口比重明显上升。改善居住条件。全面完成煤矿棚改、采沉区棚改和宝山区采沉区综合治理任务，使4.45万户棚户区居民搬入新居。实现行政区与矿区统筹协调发展。完成独立工矿区“两供一业”改造，并把“两供一业”改造与独立工矿区搬迁改造相结合，与采沉区综合治理相结合，与行政区基础设施建设、功能完善相结合，与未来产业发展承载相结合，大力推进基础设施和服务功能进一步完善，管理水平进一步提高，使矿区面貌发生重大变化。改善农村生活条件。全面完成农村泥草（危）房改造，乡镇、村屯主街路硬化率达到100%。乡镇和中心村自来水普及率达到100%，农村居民饮水合格率达到100%。完成美丽乡村建设任务。提升社会事业发展质量。实现义务教育均衡发展，普及高中阶段教育。基本建成现代公共文化服务体系，社会保障、医疗卫生等公共服务体系更加健全。

全面建成小康社会，加快转型发展步伐，建设活力幸福秀美文明双鸭山，目标鼓舞人心，蓝图催人奋进。这既是中央、省委省政府和市委的要求，更是全市人民的热切期盼。在前进的道路上，虽然面临着诸多困难和挑战，但只要我们众志成城、迎难而上，合力攻坚、奋发作为，就一定能够实现既定目标，与全省、全国同步全面建成小康社会！只要我们坚定不移地转方式、调结构，重创新、聚动能，就一定能够在转型发展上迈出坚实的步伐！只要我们始终把人民放在心中最高的位置，敢于负责、勇于担当，就一定能够让全市人民过上更加幸福美好的生活！

三、2017年主要工作任务

2017年，是实施“十三五”规划的重要一年，也是我市脱困转型、振兴发展的关键一年。政府工作总体要求是：全面贯彻中央、省委经济工作会议精神和市委十一届一次全会精神，以习近平总书记系列重要讲话特别是对我省两次重要讲话精神为根本遵循，坚持稳中求进工作总基调，适应把握引领经济发展新常态，把发展作为第一要务，以提高发展质量和效益为中心，以深化改革和创新驱动为动力，以增进人民群众福祉为出发点和落脚点，全面做好稳增长、促改革、调结构、惠民生、防风险各项工作，加快经济社会发展步伐，奋力创造脱困转型、振兴发展新业绩，稳步推进活力幸福秀美文明双鸭山建设取得扎实成效。

综合考虑，今年地区生产总值增长预期目标为6%，规模以上工业增加值增长8%，固定资产投资增长10%以上，一般公共预算收入增长8%，社会消费品零售总额增长8%，城镇和农村常住居民人均可支配收入分别增长8%和6%，节能减排完成省下达指标。上述目标是在客观把握我市经济企稳向好实际、综合分析省内同类城市发展趋势和我市预期增长因素的基础上确定的，既体现了中央稳中求进工作总基调和省委经济工作会议的要求，又满足了我市全面建成小康实现“两个翻一番”目标的客观需要，既积极稳妥，又鼓舞人心，也有利于激发各方面工作潜力和动能。实现上述目标，我们将努力做到“七个寻求新突破、一个确保”：

（一）在提高发展质量和效益上寻求新突破。一是扩大增量，全力推进产业项目建设。确保龙煤天泰10万吨芳烃、集贤三聚华本LNG、集贤北大仓现代农业产业园、集贤中纺粮食储运及深加工等45个项目竣工投产，力争神华宝清2×60万千瓦电厂首台机组实现并网发电。加快集贤华本20万吨费托合成油、宝清宝兴原有机果蔬产业园等40个项目建设。推进双矿2×35万千瓦低热值煤热电、宝清万里润达200万吨粮食深加工、尖山双紫缘1.2万吨紫苏精深加工全产业链、饶河百利10万吨矿泉水等65个项目开工。加快经开区60万吨煤制烯烃、40万吨煤制乙二醇、山东阜丰300万吨玉米深加工等21个项目前期工作。全年建设500万元以上产业项目150个，其中5000万元以上项目22个、亿元以上项目43个；完成投资70亿元。谋划储备500万元以上产业项目150个。同时，继续大力抓好招商引资工作。要深入做好项目策划生成工作，强化项目论证和包装，拿出一批市场对接成功率高的成熟项目。要进一步完善客商数据库和招商项目数据库建设，搭建招商引资信息化平台，通过“互联网+”和大数据等现代化技术的运用，提高招商引资的现代化水平。要创新招商方式，更多地采取专项招商、定向招商、以商招商、中介机构招商等新的、有效的、务实的招商方式，进一步提高招商引资的针对性和时效性。二是激活存量，充分发挥规上工业在稳增长促发展中的拉动作用。存量企业是我们重要的家底，是稳增长促发展的重要依托。要针对全市规上工业存在的问题，制定扭亏增盈、企稳向好的实施方案，对全市规上工业企业进行全面排查梳理，对存在困难的企业要结对帮扶，一企一策，精准施策，从供需两端发力，全力帮助企业解决资金、技术、供需对接、要素配置等各类重点难点问题，尽快帮助企业渡过难关、企稳回升、焕发新的生机。对于启动无望的停产企业，要加大引联力度，通过嫁接重组、技术改造等办法，加快把闲置的存量转化为新的增量。力争集贤威克特30万吨生物饲料加工、友谊龙粮30万吨粮食仓储及玉米特强粉深加工等盘活存量项目建成投产。全年必保盘活停产企业10户以上。三是强化载体，加快市经开区建设步伐。要进一步理顺经开区建设管理体制，完善内设机构，全面强化项目招商、基础设施建设、为企业服务和融资职能。以市经开区为引领，积极探索跨区域合作共建共享园区模式，推动集约化、规模化、链式协同发展。明确并落实支持产业发展的优惠政策和奖励措施。今年要在基础设施建设和项目引进上有新的突破。市经开区力争落户5000万元以上产业项目10个以上（其中亿元以上项目5个），完成招商引资到位资金10亿元以上。四是扩量提质，大力发展民营经济。民营经济增长至少要达到7%以上。这就要拿出真办法、硬措施，加大政策支持力度，加快推动民营经济扩量增速、提档升级、创新发展。全年新增小微企业200户、规上企业10户以上，推动20户企业建立现代企业制度。五是聚势发力，推动区域一体发展上水平。按照要事共商、发展共谋、成果共享的原则，建立健全市矿、地方与农垦系统、地方与森工系统“区域一体发展”协调联动机制，推动双方在做好“三篇大文章”、做实“四头四尾”等方面进行卓有成效的合作，共同创造转型发展新成果。全力支持双矿打好改革脱困攻坚战，持续抓好双矿困难职工救助工作，完成第二批双矿分流人员转岗安置和就业创业服务工作。

（二）在培育新的经济增长点上寻求新突破。要在以下七个有基础优势和发展潜力的产业方向上下足功夫。一是畜牧产业。以“两牛一猪”为重点，大力推进规模养殖、屠宰加工，加快把我市打造成高品质畜产品基地。在饶河引进建设深圳黑土地6000头丹麦种猪繁育、饲养及无抗冷鲜肉加工产业链项目，在尖山建设谷实农牧10万头生猪养殖项目，在集贤建设青岛康宏利10万头生猪养殖项目，在宝清建设中顺万头肉牛养殖项目，加强与广东温氏、深圳金新农、北京大北农、台湾嘉一香等企业洽谈、协商，尽快引进在我市落户。二是林业产业。以林中种植、养殖和林产品加工为重点，打造绿色林产品基地。开工宝清万亩富硒人参产业园，加快集贤5万亩蓝靛果繁育种植加工，全市新增果林3000亩，新增挂袋木耳100万袋，新增中草药1.2万亩。林业产值增长16%。同时，大力推动四方台满源、尖山瑞丰、宝山坤德食用菌基地加快建设，完成岭东双瑞花菇基地建设，加快岭东双瑞食用菌精深加工项目建设。三是电子商务产业。按照“模式升级、发展增速、产业壮大”的思路，打造一批本地电商平台、一批年销售额超百万的电商企业和电商团队，推动电商产业产生更大的价值和效益。与新华网合作建成黑龙江省电商基地总部、与深圳报业集团合作建成双鸭山市特色农产品电商平台、与中国农科院信息化所合作建成智慧农业平台、与山东寿光蔬菜集团合作建成大宗农产品交易平台等本地电商平台，有效地促进双鸭山本地平台拓展与升级。四是旅游产业。实施“两带”牵动，叫响“中国·黑土湿地之都”和“乌苏里江·界江风情”品牌，推动旅游产业提升层次、加快发展。继续推动与上海景域集团、中促旅、中旅联盟等企业务实合作，力求引入资金建设一批旅游项目。加快乌苏里江国家湿地公园二期、赫哲族民俗文化风情旅游小镇、大顶子山旅游综合开发、宝清梨树山庄旅游综合开发等景区建设，全市旅游收入增长30%以上。五是冰雪产业。制定冰雪产业发展规划，推动冰雪产业与体育产业、教育普及和文化活动相结合，谋划实施一批中高端冰雪产业项目，谋划举办一批冰雪活动和冰雪赛事，把“冷资源”转化为“热经济”。扩大青山滑雪场、冰雪乐园的市场运营能力，组建市冰球队、速滑队，充分利用市冰上体育运动中心，承载和推动专业性和群众性的体育运动健康发展。六是健康养老产业。突出天然氧吧、绿色食品、中医药养生特色，吸引社会资本投资养老产业，注重利用闲置资产，重点发展规模较大、功能配套、医养结合、绿色生态的养老产业项目，完成集星养老服务中心项目建设，推进宝山安康养老、尖山福泽康年、宝清阳光老年公寓等项目投入使用。七是现代服务业。推动银行创新信贷模式和产品，支持保险证券业发展，加快发展新型金融、中介服务等现代服务业发展。

（三）在加快发展现代农业上寻求新突破。一是大力调整农业种植结构。减玉米、强大豆、增水稻、杂粮和果菜，扩大优质高效作物种植面积，大力实施农业“三减”耕作模式，提高农产品品质。玉米、水稻、大豆种植比例由上年的62.7:20.4:16.9调整为59.7:22.8:17.5，农业“三减”面积达到60万亩。二是扩大绿色有机食品基地。以42个“互联网+农业”高标准示范基地建设为重点，实现基地农产品检测和质量跟踪追溯全覆盖，以此为带动，将全市绿色有机食品基地面积扩大到135万亩。狠抓品牌创建，引导企业和农民通过产权股权重组、合约使用、授权许可等方式整合绿色食品品牌，树立统一品牌形象，打造有竞争力的知名品牌。今年，重点以“宝清富硒大米”、“饶河大米”、“宝清红小豆”、“宝清大白板”、“太保胡萝卜”等地理标志品牌为依托，加快推进品牌整合，力求取得重大突破。创建水产品健康养殖示范基地。三是狠抓农业基础设施建设。加快大叶沟水库和常胜水库建设前期工作。完成饶河小佳河近期治理二期、集贤柳树河堤防、寒葱沟水库生态修复水土保持及水毁修复等工程。扎实推进农村饮水安全巩固提升工程，解决20个村屯、2万人饮水安全问题。深入实施节水增粮行动工程，新增高效节水灌溉面积2万亩。完成集贤县兴安乡和饶河县西丰镇6355公顷土地整治项目。新增各类农机具2000台套，综合机械化率达到95%。四是强化农产品市场营销。鼓励农业合作社、农民、中介机构参加各类展会。引导和鼓励农业高标准种植示范基地、农业企业、农民合作社自建平台或利用第三方平台，开展线上线下销售，搞好订单销售。农产品线上交易额增长25%。五是深化农业改革。按照“企业+合作社+农户”经营模式，培育新型农业经营主体，大力发展规范化合作社和家庭农场。落实“三权分置”办法，发展适度规模经营，新增土地规模经营面积10万亩，达到253万亩以上。加快推进农村土地确权工作，确保年内完成全部确权任务。选择试点村，推进农村集体产权制度改革，探索土地股份合作新模式。

（四）在深化改革扩大开放上寻求新突破。这是我市转型发展的动力源泉。一是突出抓好重点领域改革。持续推动行政审批制度改革。坚持“多取消、审一次、真备案”，进一步精简审批事项，提高“放”的含金量、协同度，推进“扩权强区”、“扩权强县”，确保接得住、管得好。动态调整权力清单、责任清单、中介服务清单，着手建立负面清单。明确和简化审批及公共服务流程。强化事中事后监管，推动“双随机、一公开”监管方式全覆盖。继续推进商事制度改革，推动企业注册登记便利化。深化公共资源市场化配置改革，将工程建设招投标、土地使用权和矿业权出让、国有资产交易、政府采购等纳入全市统一的公共资源交易平台。推进城市管理综合执法改革。调整经济综合管理部门职能和内设机构，强化发展职能和服务职能。事业单位改革从控编减编做起，加快制定配套政策，大力度向前推进。抓好从事实质性生产经营活动的“三权合一”事业单位转企改制。完成厂办大集体改革。二是推进以对俄为重点的对外开放。发挥饶河口岸作用，完成饶河口岸查验设施、综合海关监管库等项目建设，进一步优化口岸通关环境、完善口岸整体功能。做大中俄互市贸易区。强化市政府对口岸的统筹管理能力和外贸服务功能。立足我市地理区位优势，坚持“出口抓加工、进口抓落地”，推进宝山“爱喜爱”宠物食品出口基地建设、四方台昊天胡萝卜出口基地建设，完成饶河萨沙绿色蔬菜出口基地和岭东双瑞花菇出口基地建设。重启中俄国际文化物流经贸产业园建设，完善四达中俄国际贸易中心功能，大力发展对俄物流产业，加大对俄贸易量，提高对俄经贸的质量和水平。三是扩大以双深合作为重点的对外交流合作。确保深粮中信粮食仓储深加工项目竣工投产，推动与深圳贝特瑞公司签约的新材料（石墨）产业园、与深农黑龙江海吉星公司签约的现代农业产业园在市经开区落地实施，完成1.8万亩的黑龙江海吉星现代农业产业园农产品生产基地建设。加大华润集团啤酒生产和双鸭山和富支付科技发展有限公司等各类项目的推进力度，尽快取得实质性进展。在原有工作基础上，继续加大双深合作力度，把握住难得的机遇，寻找新的合作机会和伙伴，力争引进一些战略投资者和科技创新型项目，坚持引资与引技、引智、引模式相结合，进一步推动双深合作向宽领域、深层次持续迈进。同时，在双深合作中，我们还要不断解放思想、更新理念、学习方法、培养锻炼干部。此外，我们还要进一步推动与“珠三角”、“长三角”、“京津冀”等发达地区的交流与合作，推动我市对外开放整体上层次、上水平。

（五）在实施创新驱动发展战略上寻求新突破。突出推进以科技创新为核心的全面创新，加快新旧动能转换，提升发展核心竞争力。一是大力推动科技成果产业化，培育发展新动能。坚持引成果、引技术、引人才并举，加快与高等院校、科研院所建立科技成果产业化创新发展联盟，推动高校院所在我市建立研发中心、中试基地、工业化试生产基地，推动省内外科技成果在双产业化。今年每个县区至少要与10个高校院所建立创新发展联盟，形成常态化、规范化、有实质内容的合作机制。全年力争引进科技成果50项，实现产业化20项；新增科技型企业15户。要依托创新发展联盟，策划生成有科技含量、有供给优势、有国内市场总需求空间的项目40个以上。以深入实施科技型企业三年行动计划为牵动，推动企业成为创新成果转化的主体。二是加快创新创业平台建设，推动大众创业万众创新。建设完成集交流展示、示范引领、平台服务、电子商务、创新孵化、培训中心和物流中心等功能于一体的双鸭山“双创”孵化中心，并整合大学生就业创业服务职能和科技成果转化职能于一体，把中心建设成新型经济业态综合性产业园区。每个县区都要建设完成电子商务创业园区等平台，并完善网络化配套服务体系。加快公共就业服务平台建设，推动大学生、城镇转移就业职工、农民、专业技术人员、退伍军人等重点群体运用新模式、适应新业态、进入新领域创新创业，形成大众创业、万众创新蓬勃之势。实现城镇居民成功创业2000人，农民成功创业3000人，城镇新增就业2万人。三是建设智慧城市，推动城市治理能力现代化。要通过顶层设计，打通信息“隔离墙”、打破信息“孤岛”和数据分割，高度重视并整体规划全市的信息化、网络化、数字化建设，制定出台“智慧城市建设方案”和“互联网+行动实施意见”，重点在智慧政务、智慧公共安全、智慧农业、智慧医疗及“互联网+”的若干领域进行整体规划，分清主次和轻重缓急，分步实施。今年在顶层设计和总体规划的基础上，要选择几个重点领域进行实质推动。我们要经过强力推动和统筹整合，力争使我市智慧城市建设和“互联网+”行动实现创新发展。

（六）在城市建设发展上寻求新突破。以“一创两建”为牵动，以高标准规划为引领，以完善功能、便民利民、提升品位为方向，筹措资金33.7亿元，推进实施“六大工程”，为创建全国文明城市打造良好基础。一是城区道桥建设工程。开工建设城市南环路、建设路立交桥，启动建设中心站立交桥，完成北外环路建设，做好云峰山隧道前期工作。同时，要打通主城区“瓶颈路”和“断头路”，消除所有道路“疮疤”，主城区和各县区在提高质量的前提下，全面完成背街巷道综合整治。二是老旧小区修缮改造工程。老旧小区修缮改造，直接关系到老百姓的生活质量，是我们必须解决的重大民生问题。在财力紧张的情况下，今年我们下决心拿出1亿元，把主城区剩余的73个小区579栋住宅楼全部改造完毕，让近10万名市民的生活质量得到重大改善。同时，要改造主城区老旧供热管网50公里以上。启动独立工矿区“两供一业”综合改造。启动推进城市天然气置换工作。三是城市供排水工程。完成主城区58座供水二次加压泵站改造，取消主城区供水二次加压费，切实降低老百姓用水负担。完成7.75公里老旧小区供水支线管网改造和4.14公里供水管网优化工程。完成窑地路、马鞍山路供水改造工程，启动向阳排水明渠改造工程。四是园林绿化工程。完成云峰山公园建设，对益寿山公园进行修缮，把云峰山公园和益寿山公园连成一个整体，为市民打造设施完备、功能完善、环境优美的休闲锻炼场所。同时，要加快北秀公园维修改造，实施重点街道、广场、节点的绿化美化亮化。五是公共服务工程。建设完成第三十二中学、第二中学、第四中学、马鞍山小学、铁西小学等5所中小学校。建设完成市精神病防治院、市传染病医院，加快建设市老年人精神病福利院，开工新建市图书馆，建设完成市图书馆南市区分馆。加快市人民医院内部装修和设备安装调试进度，力争5月份投入使用，让民众关注已久的医疗设施尽快为民众所用。六是环境保护工程。全面系统规划安邦河水系的保护治理，用生态化的办法从根本上解决水质污染问题，开工建设安邦河绿色生态长廊工程，启动安邦河上游水系生态环境修复保护工程前期工作。马蹄河水污染综合治理工程建成投入使用。对域内所有河流推行“河长制”，启动保护母亲河——安邦河行动，动员全社会保护母亲河。完成双鸭山主峰山体修复一期工程。开工建设城市生活垃圾处理工程、北城区垃圾转运站及配套设施。加大大气污染治理力度，确保建龙公司干熄焦项目竣工投产，完成取缔小锅炉、淘汰黄标车的目标任务。加快公厕改造速度。在实施“六大工程”的同时，加快推进全市重要交通基础设施建设。开工建设集当公路双鸭山至宝山段、同哈公路友谊至集贤段，加快双鸭山至宝清高速公路前期工作。全力保障牡佳客专途经双鸭山段完成年度建设任务。完成高铁站和客运中心建设的前期准备。积极推进佳木斯机场迁建，宝清、饶河机场前期工作。

（七）在保障改善民生上寻求新突破。始终把保障和改善民生作为发展经济的根本目的，全面加大工作力度，切实增进民生福祉。一是全面完成脱贫攻坚任务。坚持“六个精准”和“五个一批”，压实压靠责任，强力推进，确保饶河县摘掉国贫县帽子，完成全市最后1440人的脱贫任务。二是加大安居工程建设力度。竣工煤矿棚改住宅4571户。全面完成27768户采沉棚改任务。全力争取国家政策支持，启动宝山区采沉区综合治理工程。启动光明小区二期工程。改造农村泥草（危）房4867户。三是提升社会保障水平。合理适度提高机关事业单位和退休人员基本养老水平，做好城镇职工基础养老金全国统筹实施前准备工作，确保改革举措及时落地。整合城镇居民医疗保险和新农合，建立统一的城乡居民基本医疗保险制度。调整城镇职工和城镇居民医疗保险待遇标准，将大病医疗保险调整为大额医疗保险，扩大医疗保险普惠面。继续提高社会救助保障标准，合理提高城乡低保和特困人员救助供养水平。四是推动教育公平优质发展。在新建5所中小学的同时，新建和改扩建公办幼儿园6所，支持幼儿教育健康发展。推动四个区和集贤、友谊、饶河三个县加强学校的软硬件建设，切实提高教学质量和信息化建设水平，达到国家均衡教育验收标准，全市全面完成均衡教育发展的目标任务。积极支持职教集团改革发展。推动考试招生制度改革实践探索。五是抓好医药卫生工作。推进城市公立医院综合改革，扩大并完善医联体和分级诊疗模式，全面执行药品集中采购制度，降低就医负担。推进医药物流体系建设，支持药品集中配送。加强公共卫生服务，提高医疗质量和水平。城市社区卫生服务人口覆盖率达到90%，40家公立乡镇卫生院达到国家建设指导标准。加强疾病预防控制工作，维护人民群众健康权益。六是加快文体事业发展。实施文化惠民工程，完成6个社区“三室”建设。推动文化艺术机构和中小学对接，实现艺术教育、艺术辅导、艺术欣赏进课堂。推动艺术创作生动反映双鸭山市在脱困转型过程中出现的新风尚、新典型、新面貌。继续开展“结对子、种文化”活动，不断丰富群众文化生活。推动全民健身运动深入开展。建成并投入使用市全民健身综合馆。推进“三大球”和冰雪运动项目进入全市所有学校。

（八）确保社会安全稳定。安全生产、社会稳定事关改革发展大局，事关人民幸福安康，必须紧抓不放、长抓不懈。一是全力抓好安全生产。全面贯彻《中共中央国务院关于推进安全生产领域改革发展的意见》，把安全生产“党政同责、一岗双责、齐抓共管、失职追责”落实落靠，强化属地管理。实行安全生产网格化监督管理制度，建立层级责任清单、现场检查事项清单和隐患排查整改清单，创新管理机制，提高安全管理水平。全面抓好煤矿安全生产，严厉“打非治违”，严格整改隐患，遏制重大事故发生。加大非煤矿山、危化品生产运输企业、道路交通、建筑施工、食品药品、消防、森林防火、特种设备、人员密集场所等行业领域安全隐患排查治理力度，确保安全生产形势持续稳定。二是推进“平安双鸭山”建设。完成市应急联动指挥平台建设，提升社会安全、应急处置、民生求助水平。落实省政府提出的公安系统“六大建设工程”任务，严厉打击各类违法犯罪活动，完善打防控体系，实现治安、刑事案件发案双下降，进一步提升人民群众安全感和满意度。三是加强信访工作。严格落实信访工作责任制，坚持工作重心下移、强化首接首办责任。高度重视初信初访，全力把矛盾化解在初始状态、问题解决在基层，切实减少存量、严控增量。加大积案化解力度，做到诉求合理的解决到位，诉求无理的思想教育到位，生活困难的帮扶救助到位，行为违法的依法处理，确保信访稳定、社会稳定。

四、切实加强政府自身建设

推进活力幸福秀美文明双鸭山建设，完成全年工作任务，是大目标、硬任务。要求各级政府必须增强责任意识、担当意识，主动适应新形势、谋划新举措、施展新作为，不断提高工作能力水平和实效。

（一）着力建设专业化政府。好学才能上进，好学才有本领。建设专业化政府，既是建设现代政府的内在要求，也是新形势下推动转型发展、全面建成小康社会的迫切需要。各级政府和政府系统广大干部要把加强专业知识学习摆在突出重要位置，坚持干什么学什么、缺什么补什么，有针对性地加强学习，特别要加强对新产业、新业态、新技术、新模式的学习和研究，努力在实践中增长才干，加快知识更新、优化知识结构，着力避免陷入“少知而迷、不知而盲、无知而乱”的困境，着力克服本领不足、本领恐慌、本领落后的问题。要继续办好“双鸭山发展讲坛”，巩固好政府系统专业知识专题学习制度，使学习专业知识成为一种常态、一种自觉、一种风气，不断提升运用专业思维、专业素养、专业方法干工作、搞建设、促发展的能力和水平，使我们的能力和水平能够适应带领全市人民抓转型、建小康的工作需要。

（二）着力建设法治政府。各级政府必须强化法治观念，认真实施法治政府建设五年规划，加强法治政府建设，做到权责法定、依法行权。要把公众参与、专家论证、风险评估、合法性审查和集体讨论决定作为重大决策必经程序，科学决策、民主决策、依法决策。充分发挥法律顾问的重要作用，有效防范决策和行政法律风险。自觉接受人大及其常委会法律监督、人民政协民主监督、社会各界和人民群众监督，认真办理好人大代表议案、建议和政协委员提案。全面推行政务公开，以公开促公正。大力规范执法行为，使公平文明执法成为执法的价值取向。坚持依法治政与以德治政相结合，以德治促法治。在城市建设管理、环境保护、历史文物等方面提出地方立法建议，强化依法治市。推动各级政府领导干部强化法治思维，提高运用法治方式开展工作、解决问题、推动发展的能力。抓好“七五”普法，引导广大群众自觉守法、遇事找法、解决问题靠法，让依法办事蔚然成风。

（三）着力建设诚信政府。建设诚信政府，是建设法治政府的内在要求，也是从根本上优化发展环境的必然要求。通过与大公国际集团合作，启动建设社会信用体系。各级政府要把建立政府诚信体系摆在重要位置，把政府运行的各个环节置于诚信规范约束之下。要使守信践诺成为政府的忠诚品格，切实强化契约精神，严格依法依规办事、按政策办事，做到言必行、行必果，决不做出尔反尔、言而无信的事情。要把创造公平宽松环境作为工作的重要着力点，严厉查处不作为、乱作为、慢作为行为，除特殊情况外，未经批准，一律不得开展涉企检查，确保企业正常生产经营“零干扰”，使政府权力部门与企业的关系真正能够做到“需要你时你在身边，不需要你时你在天边”。要优化政商关系，在“清”的基础上，真心亲商安商富商，让包括企业在内的所有价值创造者在双鸭山这片土地上安心投资、舒心发展。

（四）着力建设廉洁政府。全面从严治党，要求我们必须全面从严治政、廉洁从政。各级政府领导班子要始终把政府廉洁建设的主体责任扛在肩上、抓在手上，做到廉洁建设与政府工作同步研究、同步推进、同步落实。始终牢记权力是人民赋予的“权力观”，为民用权、依法行权、廉洁治权。巩固“三严三实”专题教育成果，弛而不息纠正“四风”。始终坚持艰苦奋斗，厉行勤俭节约，大力压缩一般性支出，保障民生等重点支出，确保“三公”经费“只减不增”。切实加强对重大事项决策、大额资金使用、政府投资项目、公共资源交易、资本运作等重点领域和关键环节的廉洁风险防控，织密扎紧制度的笼子。切实加强审计监督和行政监察。加大案件查办力度，做到有案必查、有贪必肃。

（五）着力建设团结和谐有战斗力的政府。团结出战斗力，和谐出凝聚力。各级政府领导班子要切实增强党性观念和全局意识，全力维护、倍加珍惜建立在党性原则和共同事业追求基础上的团结和谐，切实把各级班子打造成极具凝聚力、战斗力的坚强领导集体，建设成谋大事、干实事、求实效的工作团队。要严格执行民主集中制，坚持集体领导与个人分工负责相结合，做到民主基础上的集中和集中指导下的民主相结合。努力营造讲纪律、讲规矩、讲程序，集体力量凝聚、个人心情舒畅的良好氛围。要从严从实抓班子带队伍，坚决整改“庸懒散慢粗”，努力做到坚持问题导向，善于解决难题，求真务实，敢于担当。要发扬优良的工作作风，经常深入基层，问计于民、问需于民，做到推进工作在一线、解决问题在一线、创造业绩在一线。要始终把人民群众放在心中和脑中，深怀为民之心、恪守为民之责，切实做到忧民、爱民、为民、惠民。今年，市政府共谋划了50项民生实事。这些民生实事，既有群众最期盼解决的急事难事，也有事关群众长远福祉的大事要事，已经全部写在了《政府工作报告》当中。对此，全市各级政府和广大干部要进一步埋头实干、埋头苦干，千方百计抓好落实，切实完成好《报告》中确定的各项目标任务和民生实事，以工作实绩回报人民的信任和厚望！

各位代表，推进活力幸福秀美文明双鸭山建设，完成全年工作任务，使命光荣、责任重大。我们坚信，只要我们认真、坚决地贯彻落实党中央和省委省政府的决策部署，紧紧依靠市委的坚强领导，抢抓机遇、攻坚克难、团结拼搏、开拓创新、善作善成，就一定能够圆满完成今年各项工作任务，就一定能够开创脱困转型、振兴发展的新局面，就一定能够如期实现全面建成小康社会的伟大目标！
